# Effect on cardiovascular outcome of sodium-glucose co-transporter-2 (SGLT2) inhibitors among cancer patients treated with anthracycline: a systematic review and meta-analysis

**DOI:** 10.3332/ecancer.2025.1844

**Published:** 2025-02-12

**Authors:** Chalothorn Wannaphut, Phuuwadith Wattanachayakul, Sakditad Saowapa, Ben Ponvilawan, Manasawee Tanariyakul, Jakrin Kewcharoen, Pitchaporn Yingchoncharoen, Thanathip Suenghataiphorn, Noppawit Aiumtrakul, Jared Acoba

**Affiliations:** 1 Department of Medicine, John A. Burns School of Medicine, University of Hawai’i, Honolulu, HI 96813, USA; 2 Department of Medicine, Albert Einstein Healthcare Network, Philadelphia, PA 19141, USA; 3 Sidney Kimmel Medical College, Thomas Jefferson University, Philadelphia, PA 19107, USA; 4 Department of Internal Medicine, Texas Tech Health Science Center, Lubbock, TX 79430, USA; 5 Department of Internal Medicine, University of Missouri–Kansas City School of Medicine, Kansas City, MO 64108, USA; 6 Division of Cardiology, Department of Medicine, Loma Linda University Health, Loma Linda, CA 92354, USA; 7 Faculty of Medicine Siriraj Hospital, Mahidol University, Bangkok 10700, Thailand; 8 University of Hawai’i Cancer Center, Honolulu, HI 96813, USA; 9 Queen’s Medical Center, Honolulu, HI 96813, USA

**Keywords:** SGLT2 inhibitors, cardiovascular outcomes, anthracycline, cancer, meta-analysis

## Abstract

**Background/objectives:**

Sodium-glucose-co-transporter-2 (SGLT2) inhibitors have shown benefit in reducing cardiovascular disease outcomes in diabetes patients. Anthracycline therapy is associated with a risk of cardiomyopathy. However, the impact of SGLT2 inhibitors in the prevention of cardiomyopathy and heart failure in cancer patients undergoing anthracycline treatment remains unclear. Thus, we conducted a systematic review and meta-analysis to explore the effect of the prevention of cardiovascular outcomes in patients with cancer and diabetes who had received anthracycline therapy.

**Methods:**

We systematically reviewed Medline and EMBASE databases from inception to January 2024 for studies focusing on cancer patients with a history of anthracycline therapy. Eligible studies had to report relative risk (RR) with 95% confidence intervals (CIs) for the clinical endpoints of mortality outcomes and the risk of heart failure exacerbation, comparing cohorts with and without SGLT2 inhibitor use.

**Results:**

Our study included four retrospective cohort studies in the meta-analysis (*n* = 6,708, 24% received SGLT2). There was significantly lower all-cause mortality in the SGLT2 inhibitors group (pooled RR of 0.52, 95% CI 0.35–0.77, *I*^2^ 64%). However, there were no differences in the risk of heart failure exacerbation (pooled RR of 0.67, 95% CI 0.39–1.14, *I*^2^ 17%).

**Conclusion:**

Our study found that anthracycline-treated cancer patients using SGLT2 inhibitors experienced lower all-cause mortality compared to the control group. A randomised clinical trial is necessary to further elucidate these findings.

## Introduction

Anthracycline is a cornerstone in the treatment of multiple solid organ and hematologic malignancies. Unfortunately, it is also associated with cardiotoxicities including heart failure, cardiomyopathy and arrhythmia. These adverse effects can markedly diminish patients' overall prognosis [1–3]. Sodium-glucose co-transporter-2 (SGLT2) inhibitors have emerged as a promising therapeutic class in the management of heart failure, offering benefits such as reduced mortality, fewer heart failure hospitalisations and improved cardiac function [4–8]. The observed benefits lead to growing interest in exploring their use beyond typical diabetes or heart failure therapy, notably in the treatment of cardiotoxicity in oncology patients.

However, the application of SGLT2 inhibitors in the context of anthracycline-induced cardiotoxicity remains limited. While preliminary evidence suggests potential protective effects, comprehensive clinical data are scarce. This gap underscores the need for a systematic evaluation of existing studies to formulate clearer insights into this specific scenario.

To address this knowledge gap, we aim to conduct a systematic review and meta-analysis focusing on the use of SGLT2 inhibitors in patients undergoing anthracycline therapy. By collating and analyzing data from various clinical trials and observational studies, we intend to assess the impact of SGLT2 inhibitors on mortality outcomes and HF exacerbation in this population. In this research, our goal is to enhance therapeutic approaches and improve long-term outcomes for patients with malignancies undergoing anthracycline therapy.

## Methods

### Literature search strategy

**Figure 1.**
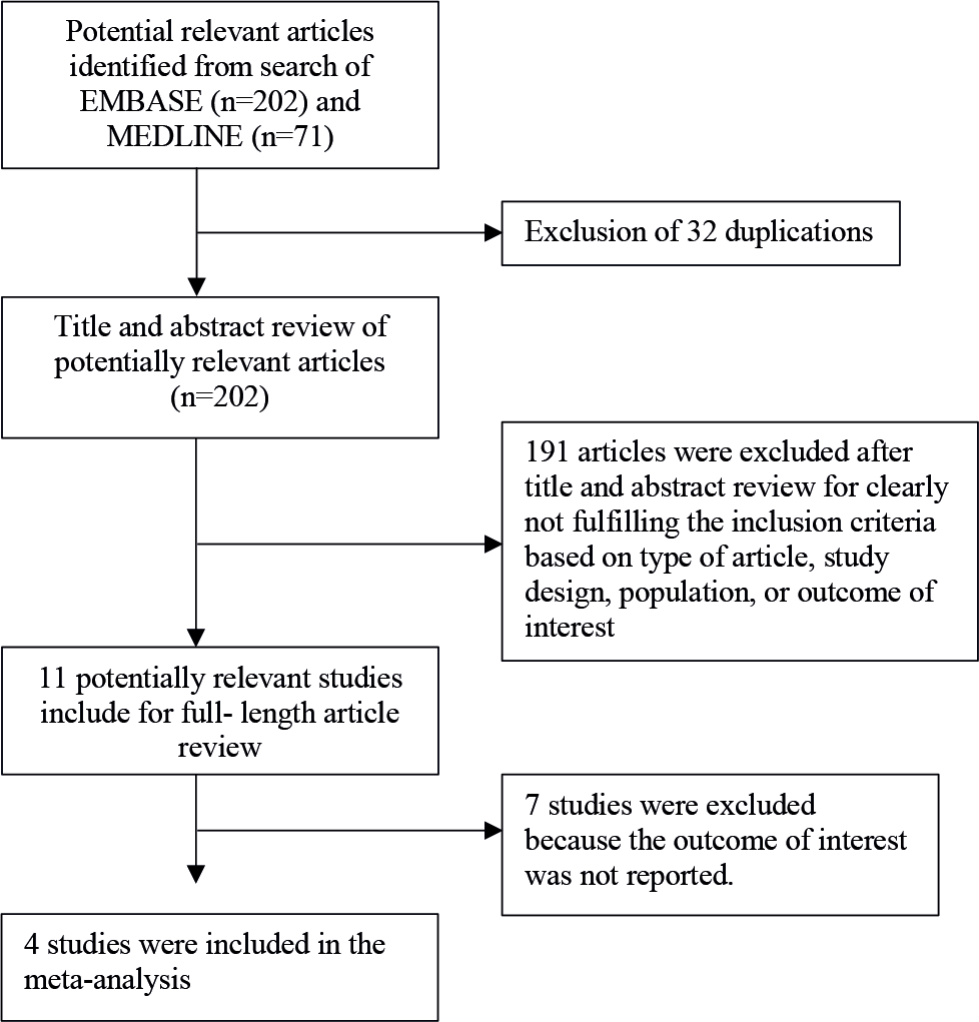
Flow chart of literature review process.

Two investigators (C.W. and P.W.) independently searched for published articles indexed in MEDLINE and EMBASE databases from inception to 14 January 2024, using the search strategy that included the terms for SGLT2 inhibitor, anthracycline and cardiac outcomes. The search strategy is available as Supplementary data 1. References of the included studies were also manually reviewed for additional eligible studies. This study was undertaken under the Preferred Reporting Items for Systematic Reviews and Meta-Analyses statement, which is available as Figure 1.

### Selection criteria

To be eligible for the meta-analysis, the study must be a cohort study that investigated survival and clinical outcomes in cancer patients treated with anthracycline between SGLT2 inhibitor use and without SGLT2 inhibitor use. Eligible cohort study must consist of patients with SGLT2 inhibitors and comparators without SGLT2 inhibitors. The eligible study must also provide the magnitude of association, which could be odd ratio (OR), relative risk (RR), hazard ratio (HR), incidence rate ratio (IRR) or standardised incidence ratio along with its corresponding confidence interval (CI).

All retrieved articles were reviewed independently by the two investigators (C.W. and P.W.) for their eligibility. The last investigator (CW) reviewed all the included studies again to ensure that the inclusion criteria were met and also served as the deciding vote when different determinations of study eligibility were made by the first two investigators. Newcastle–Ottawa quality assessment scale was used to assess the quality of the included cohort studies [9]. This scale evaluates the quality of the included studies in three areas including recruitment of participants, comparability between the groups and ascertainment of the outcome of interest for cohort study.

### Data extraction

A standardised data collection form was used to extract the following information: last name of the first author, study design, year(s) of study, country of origin, year of publication, sample size, baseline characteristics of participants, methods used to identify and verify the acute heart failure, hospitalisation confounders that were adjusted and adjusted effect estimates with 95% CI. This data extraction was independently performed by the same two investigators (C.W and P.W.) to minimise error. Any discrepancies found in the case record forms were resolved by referring back to the original articles.

### Statistical analysis

Review Manager 5.3 software from the Cochrane Collaboration was used for data analysis. Point estimates and standard errors were extracted from individual studies and were combined using the generic inverse variance method as described by DerSimonian and Laird [10]. A random-effect model, rather than a fixed-effect model, was used because the included studies were of different methodologies and background populations. OR or HR of cohort study was used as an estimate for RR to calculate the pooled RR along with RR of cohort studies. Statistical heterogeneity was assessed using Cochran's *Q* test. This statistic is complemented with the *I*^2^ statistic which quantifies the proportion of the total variation across studies that is due to heterogeneity rather than chance. A value of *I*^2^ of 0%–25% represents insignificant heterogeneity, 26%–50% low heterogeneity, 51%–75% moderate heterogeneity and >75% high heterogeneity [11]. The presence of publication bias would be assessed by visualisation of the funnel plot.

## Results

The systematic search identified 234 relevant articles (163 articles from EMBASE and 71 articles from MEDLINE). After the exclusion of 32 duplicated articles, 202 articles underwent title and abstract review. A total of articles were excluded at this stage as they did not fulfill the eligibility criteria based on the type of article, study design, participants and outcome of interest. A total of 11 articles were retrieved for full-length article review, and 7 articles were excluded at this stage as they did not report the association of interest. Finally, 4 cohort studies [12–15] with 167,907 participants (1,616 patients were on SGLT2 inhibitors) were eligible for the meta-analysis. The literature retrieval, review and selection process are shown in Figure 1. The characteristics of the included studies and their quality assessment are described in Table 1.

Briefly, among the four included cohort studies [12–15] with 167,907 participants, four studies were retrospective cohort 2 studies from the United States [13, 14], 1 studies from Canada [12] and Korea [15]. Three studies reported effect estimates as HR [12, 14, 15], and one study reported as OR [13]. Therefore, HR of the three studies, and OR of one study was used as an estimate to calculate pooled RR. All studies reported a significant mortality rate in non SGLT 2 inhibitor use. All of the eligible studies adjusted their effect estimate for any potential confounders such as demographic data and commodities.

### All cause of mortality of SGL2 inhibitor use

**Figure 2.**
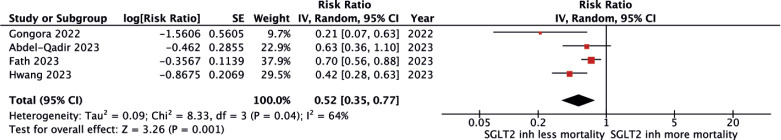
Forest plot of the meta-analysis.

A total of four cohort studies [12–15] reported lower all causes of mortality among patients using SGLT2 inhibitors than those without SGLT 2 inhibitors with a pooled RR of 0.52 (95% CI 0.35–0.77). The between-study heterogeneity was high with an *I*^2^ of 64%. Figure 2 demonstrates the forest plot of this meta-analysis.

### Risk of acute heart failure exacerbation

**Figure 3.**
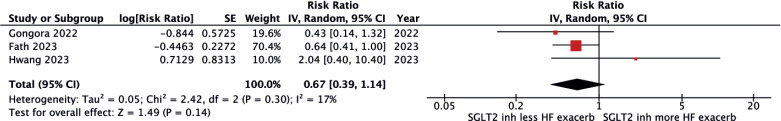
Forest plot of the meta-analysis.

A total of three cohort studies [13–15] reported the risk of heart failure exacerbation was lower in those who received SGLT2 inhibitors, though the results were not statistically significant, with a pooled RR of 0.67 (95% CI 0.39–1.14, *I*^2^ 17%). The between-study heterogeneity was high with an *I*^2^ of 12%. Figure 3 demonstrates the forest plot of this meta-analysis.

### Evaluation for publication bias

Evaluation for publication bias using visualisation of funnel plots could not be performed due to the limited number of included studies.

## Discussion

Several mechanisms of AC-induced cardiotoxicity have been proposed including oxidative stress, DNA damage, impaired iron metabolism, mitochondrial dysfunction and autophagy dysregulation [16]. Additionally, diabetes mellitus is associated with an increased risk of anthracycline-induced cardiotoxicity. Cardiac exposure to high blood glucose levels increases fatty acid and cytokines, resulting in the accumulation of fat droplets in myocardial cells and ultimately mediating cardiotoxicity [17]. Significantly, SGLT2 inhibitors exhibit noteworthy advantages in mitigating heart failure and cardiovascular mortality among individuals, irrespective of their diabetic status. To the best of our knowledge, this is the first systematic review and meta-analysis to evaluate the effect of SGLT2 inhibitors in the prevention of heart failure and cardiac outcomes in patients with cancer treated with anthracycline. Our findings indicate that patients with SGLT2 inhibitors have lower all-cause mortality compared to the non-SGLT2 inhibitors cohort with RR 0.52 with several possible explanations.

First, SGLT-2 inhibitors have demonstrated a cardiomyocyte-protective effect against anthracycline-induced cardiotoxicity through multiple mechanisms. At the molecular level, these mechanisms include the reduction of karyorrhexis and karyolysis, modulating the AMPK-mTOR signaling pathway to regulate autophagy and enhancing mitochondrial function by mitigating mitochondrial shrinkage [18]. Additionally, SGLT-2 inhibitors exhibit anti-inflammatory effects, decreasing the production of IL-1, IL-6, IL-8 and TNF-α and inhibiting reactive oxygen species formation in doxorubicin-treated cells. Regarding cardiovascular structure, function and outcomes, the utilisation of SGLT-2 inhibitors such as empagliflozin and dapagliflozin shows promise in reversing adverse effects on left ventricular ejection fraction. Empagliflozin, in particular, exhibits a reduction in markers of myocardial fibrosis, such as collagen [19]. SGLT-2 inhibitors also help rebalance intracellular sodium and calcium levels in cardiomyocytes, supporting improved left ventricular systolic function post-myocardial infarction and mitigating calcium overload caused by doxorubicin. Moreover, their diuretic effect may reduce heart failure risk associated with steroid use or fluid retention during anthracycline treatment [12]. In addition to these effects, SGLT2 inhibitors exhibit anticancer effects by modulating cancer cell metabolism, including inhibiting β-Catenin, activating AMPK and reducing ATP production, which suppresses DNA/RNA synthesis and proangiogenic factors [20].

Furthermore, SGLT2 inhibitors contribute to weight reduction through glycosuria, thereby improving insulin resistance. The reduction in body weight has been associated with a lowered risk of certain cancers, including obesity-associated breast and colon cancers [15]. However, our study shows no differences in the risk of heart failure exacerbation (pooled RR of 0.67, 95% CI 0.39–1.14, *I*^2^ 17%). The mechanism by which SGLT2i can reduce HF in patients receiving anthracycline therapy is unclear. The possible mechanisms have been explained above.

This meta-analysis carries some limitations that should be acknowledged. First, the statistical heterogeneity of the meta-analysis of all-cause mortality was moderate. Different participant characteristics were probably one of the main reasons for the variation. Second, the majority of the included studies relied on diagnosis codes from administrative databases to identify diagnoses and treatments. Therefore, the completeness of case identification, accuracy and stage of the cancer treatment, type of SGLT2 and outcome occurrences outside the database are limited. Finally, the small number of included studies in the meta-analysis could jeopardise the validity and interpretation of the funnel plot.

## Conclusion

The current study found that the SGLT2 inhibitors are associated with a lower all-cause of mortality in cancer patients treated with anthracycline but no difference in acute heart failure exacerbation. This supports conducting randomised trials testing the effect of SGLT2 inhibitors on cardiac outcomes in patients treated with an anthracycline.

## Conflicts of interest

All authors declare no personal or professional conflicts of interest, and no financial support from the companies that produce and/or distribute the drugs, devices or materials described in this report.

## Funding

None.

## Figures and Tables

**Table 1.** Baseline characteristics of studies included in the meta-analysis.

	Hwang *et al* [15]	Abdel-Qadir *et al* [12]	Fath *et al* [13]	Gongora *et al* [14]
Year of publication	2023	2023	2023	2022
Country of origin	South Korea	Canada	Multi-center	United States
Study design	Retrospective cohort	Retrospective cohort	Matching cohort	Retrospective cohort
Total number of participants	Total: 81,572 patients non-DM: 77,337 patients SGLT2i: 780 patients Non-SGLT2i: 3,455 patients	933 total patients 99 SGLT2i patients 834 non-SGLT2i patients	1,412 patients, divided into 706 patients in each Group (SGLT2i versus not SGLT2i)	Diabetes with SGLT2: 32 patients Diabetes without SGLT2: 1,807 patients
Recruitment of participants	Patients aged ≥18 years who were newly diagnosed with cancer and underwent anthracycline-containing chemotherapy between January 2014 and December 2021 from nationwide claims data from the Health Insurance Review and Assessment (HIRA) Service database of South Korea	Patients more than 65 years of age with treated diabetes and without prior HF who received anthracycline-based chemotherapy for cancer, using the Ontario Cancer Registry, treated between 1 January 2016, and 31 December 2019.	Using TriNetX Global Research Network from 2013 to 2021, patients diagnosed with cancer and receiving anthracycline therapy were identified and categorized into taking SGLT2i versus those who were not taking SGLT2i.	Patients with diabetes mellitus (DM) and cancer treated with an anthracycline in the Massachusetts General Brigham system before September 2020.
Classification of medication category	SGLT2i usage, non-SGLT2i usage, non-DM patients	SGLT2i usage, non-SGLT2i usage	SGLT2i usage, non-SGLT2i usage	SGLT2i usage, non-SGLT2i usage
Follow-up	N/A	N/A	Patients were followed for 2 years	N/A
Follow-up duration (years)	Median duration of 3.4 ± 2.3 years	1.6 years (Q1-Q3: 0.8–2.9 years	N/A	N/A
Average age of participants (years)	non-DM: 52 ± 12 years SGLT2i: 56 ± 10 years non-SGLT**2**i: 62 ± 11 years	SGLT2i: 70 non-SGLT2i: 71	N/A	SGLT2i: 60 ± 11 non-SGLT2i: 60 ± 10
Percentage of male	non-DM: 20% SGLT2i: 29% non-SGLT**2**i: 37%	SGLT2i: 35.4% non-SGLT2i: 38.1%	N/A	SGLT2i: 50% non-SGLT2i: 43%
Comorbidities	Hypertension, Dyslipidemia, Coronary artery disease	Diabetes duration; hypertension; ischemic heart disease; atrial fibrillation; chronic obstructive pulmonary disease	N/A	Cardiovascular risk factors, Obstructive sleep apnea, Coronary artery disease, Prior heart failure, Prior myocardial infarction, chronic kidney disease
Variables adjusted in multivariate analysis	Age, gender, duration of T2DM, medication history, cancer type, hypertension, dyslipidemia, coronary artery disease	Age; sex; year of chemotherapy; cancer category (breast, lymphoma, or other); median neighborhood income quintile; rural residence; diabetes duration; hypertension; ischemic heart disease; atrial fibrillation; chronic obstructive pulmonary disease; Johns Hopkins ACG System Aggregated Diagnosis Groups risk score; and the use of metformin, insulin, statins, angiotensin antagonists, and beta-blockers.	N/A	Demographics, Race, Cancer, Cardiovascular, Other medical comorbidities, Cardiovascular medications
Newcastle-Ottawa score	Selection: 4 stars Comparability: 2 stars Outcome: 2 stars	Selection: 4 stars Comparability: 2 stars Outcome: 2 stars	Selection: 4 stars Comparability: 2 stars Outcome: 2 stars	Selection: 4 stars Comparability: 2 stars Outcome: 2 stars

Abbreviation: SGLT2i, Sodium glucose transport protein 2 (SGLT2) inhibitors; DM, diabetes mellitus; T2DM, type 2 diabetes mellitus

## Supplementary data 1

### Search strategy

#### Medline

1. Exp SGLT2 inhibitors/ or exp SGLT2 inhibitor.mp.
2. Anthracycline.mp. or exp anthracyclines
3. Exp doxorubicin/ or doxorubicin.mp.
4. Adriamycin.mp. or exp doxorubicin
5. Daunorubicin.mp. or exp daunorubicin
6. Epirubicin.mp. or exp epirubicin
7. Idarubicin.mp. or exp idarubicin
8. Cancer therapy.mp. or exp antineoplastic agents
9. Exp cardiovascular diseases/ or cardiovascular outcome.mp.
10. Exp cardiovascular system system/ or exp cardiovascular abnormalities/ or cardiovascular.mp. or exp cardiovascular diseases
11. 2 or 3 or 4 or 5 or 6 or 7 or 8
12. Exp myocardial infarction/ or cardiac outcome.mp. or exp heart failure
13. Exp hospital mortality/ or exp mortality/ or mortality.mp. or exp mortality, premature
14. 9 or 10 or 12 or 13
15. Exp SGLT2 inhibitors/ or empagliflozin.mp.
16. Canagliflozin.mp. or exp canagliflozin/
17. Exp SGLT2 inhibitors/ or dapagliflozin.mp.
18. Ertugliflozin.mp.
19. 1 or 15 or 16 or 17
20. 18 or 19
21. 11 and 14 and 20

#### Embase

1. SGLT2 inhibitor'/exp odd ratio (OR) 'SGLT2 inhibitor
2. Dapagliflozin
3. Empagliflozin
4. Canagliflozin
5. Ertugliflozin
6. Bexaglifloxin
7. Anthracycline
8. Doxorubicin
9. Daunorubicin
10. Epirubicin
11. Idarubicin
12. Antineoplastic agent
13. Cancer therapy
14. Cardiovascular outcome
15. Cardiovascular disease
16. Heart failure
17. Heart infarction
18. Mortality
19. #1 OR #2 OR #3 OR #4 OR #5 OR #6
20. #7 OR #8 OR #9 OR #10 OR #11 OR #12 OR #13
21. #14 OR #15 OR #16 OR #17 OR #18
22. #19 AND #20 AND # 21
